# Factors associated with severe bacterial infection in infants between 91- and 120-days old admitted to the pediatric emergency department

**DOI:** 10.1186/s12887-025-05764-9

**Published:** 2025-05-24

**Authors:** Bei-Cyuan Guo, Han-Ping Wu, Yin-Ting Chen, Yu-Jun Chang, Chun-Yu Chen, Wen-Ya Lin, Chung-Hao Su

**Affiliations:** 1 Department of Pediatrics, National Cheng Kung University Hospital, College of Medicine, National Cheng Kung University, Tainan, Taiwan; 2 College of Medicine, Chang Gung University, Taoyuan, Taiwan; 3 Division of Neonatology, Department of Pediatrics, Chiayi Chang-Gung Memorial Hospital, Chiayi County, No. 6, W. Sec., Jiapu Rd, Puzi City, Taiwan; 4 Division of Neonatology, Department of Pediatrics, Children Hospital, China Medical University, Taichung, Taiwan; 5 Laboratory of Epidemiology and Biostastics, Changhua Christian Hospital, Changhua, Taiwan; 6 Department of Emergency Medicine, Tungs’ Taichung Metro Harbor Hospital, Taichung, Taiwan; 7 Department of Nursing, Nursing and Management, Jen-Teh Junior College of Medicine, Miaoli, Taiwan; 8 Department of Pediatric Emergency Medicine, Department of Pediatrics, Taichung Veteran General Hospital, Taichung, Taiwan

**Keywords:** Infant, 91-120 Days, Fever, Severe Bacterial Infection, Emergency department

## Abstract

**Introduction:**

Diagnosing severe bacterial infections (SBI) can be challenging, especially in infants. This study sought to identify clinical factors that could aid in predicting SBI in infants aged 91–120 days.

**Methods:**

This retrospective cohort study investigated febrile infants aged 91-120 days admitted to the pediatric emergency department (PED). This study assessed the significant predictors of clinical and laboratory data for identifying SBI in young infants.

**Results:**

This study analyzed 264 febrile infants aged 91–120 days admitted to the PED. The significant factors for infants with SBI included sex, admission weight, body temperature(BT), white blood cell (WBC) count, neutrophil percentage, and C-reactive protein (CRP) level (all *P* < 0.05). Logistic regression analysis showed that male sex, higher BT, WBC count, and CRP levels were predictors of SBI. ROC analysis identified the useful cutoff values for predicting SBI as a BT of 39.3 °C, WBC counts of 15,500/µL, and a CRP concentration of 14.4 mg/L.

**Conclusions:**

Increased BT, elevated WBC and neutrophil counts, as well as higher CRP levels, may act as predictors of SBI in infants aged 91–120 days admitted to the PED.

## Introduction

In pediatric patients, infectious diseases are a leading cause of visits to emergency departments [1, 2], with viral infections being the most common [3, 4]. Most children without an identifiable infection source typically experience self-limiting viral illnesses [5, 6]. While bacterial infections in children are relatively frequent, they can often be clinically elusive, even after a thorough physical examination, complicating diagnosis. Although severe bacterial infections (SBI) are rare, they present a significant risk of increased mortality [7–9]. Clinicians continue to face challenges in identifying febrile infants who are at the highest risk for SBI. While numerous studies have explored the risk factors and diagnostic indicators for these infections, most have primarily focused on infants younger than 90 days. However, no studies have specifically targeted infants aged 91 to 120 days [10–15]. One study examined the incidence and predictors of SBI in infants between 57 and 180 days of age [16]; however, applying these parameters to infants aged 91 to 120 days may be challenging and of limited clinical utility. This study aimed to analyze febrile infants aged 91 to 120 days admitted to the pediatric emergency department (PED) and identify relevant clinical factors that could help predict SBI in this age group.

## Materials and methods

### Patient population

This retrospective study analyzed febrile infants aged 91 to 120 days who were hospitalized in the pediatric emergency department (PED) of a medical center between 2014 and 2017. Infants were excluded if they had a history of immunodeficiency, preterm birth, comorbidities, chronic illnesses requiring antibiotic prophylaxis, or if they had received a vaccination within 48 h prior to their PED visit. All patients received their scheduled vaccinations in Taiwan. None of the patients had used fever-reducing medication or antibiotics before their arrival.

The study was approved by the Institutional Review Board and Ethics Committee of China Medical University Hospital (CMUH108-REC1-061), ensuring compliance with all relevant guidelines and regulations. Prior to analysis, data were collected, reviewed, de-identified, and anonymized. Given the anonymized nature of the dataset and the study’s scientific objectives, the Institutional Review Board and Ethics Committee waived the requirement for informed consent.

### Study designs and measurements

Fever was defined as a body temperature exceeding 38.0 °C, measured either at home or in the pediatric emergency department (PED) using any method of temperature assessment (e.g., rectal, axillary). Febrile infants were categorized into two groups: those diagnosed with severe bacterial infection (SBI) and those without. SBI was diagnosed based on the following criteria: (1) Bacteremia, identified by the presence of a pathogen in the blood through a positive blood culture; (2) Urinary tract infection (UTI), confirmed by a catheterized urine culture showing > 50,000 CFU/mL; and (3) Bacterial meningitis, diagnosed by a positive cerebrospinal fluid culture for a pathogen.

The study collected and analyzed various factors, including age, sex, gestational age (GA), delivery method, birth weight, weight upon arrival at the PED, vital signs (such as rectal or axillary body temperature, pulse rate, respiratory rate, and blood pressure) recorded during triage, length of hospital stay, and laboratory results at admission. Fever resolution was defined as the number of days from the first recorded temperature above 38 °C until it dropped below 38 °C and remained there continuously for 48 h, regardless of whether antipyretic medications, such as acetaminophen syrup, were used. At admission, laboratory tests included total white blood cell (WBC) count, neutrophil count, non-segmented bands, C-reactive protein (CRP) levels, and blood cultures. Clinical and laboratory data were compared between the SBI and non-SBI groups to identify potential risk factors for SBI in febrile infants.

### Statistical analysis

A variety of statistical tests were conducted, including the t-test, chi-square test, Fisher’s exact test, Mann-Whitney U test and logistic regression. Logistic regression was applied to analyze the influence of various factors on blood culture results. Receiver operating characteristic (ROC) curve analysis was utilized to identify the optimal cutoff values for clinical variables. This analysis evaluated variables based on sensitivity, specificity, positive likelihood ratio (LR+), negative likelihood ratio (LR-) and area under the ROC curve. Descriptive statistics were presented as percentages or medians with first and third quartiles. Group differences were reported with 95% confidence intervals. Statistical significance was considered when the p-value was less than 0.05, and all analyses were performed using IBM SPSS Statistics software (version 22.0; SPSS Inc., Chicago, IL, USA).

## Results

The study included 264 febrile infants admitted to the pediatric emergency department during the study period. All patients underwent comprehensive blood tests and cultures. The median age of the infants was 106 days, with an interquartile range (IQR) of 100–114 days, and the male-to-female ratio was 1.61. No significant differences in age, gestational age (GA), or birth weight were found between the SBI and non-SBI groups. However, the SBI group had a significantly higher proportion of males and a higher median admission weight compared to the non-SBI group, with a p-value of less than 0.05. Additionally, the SBI group exhibited higher body temperature at triage, higher peak body temperature, and longer hospital stays (all *P* < 0.05).

The SBI group also showed significantly higher white blood cell (WBC) counts, neutrophil percentages, and C-reactive protein (CRP) levels compared to the non-SBI group (all *P* < 0.05). The incidence of positive blood cultures was higher in the SBI group (4.2%) compared to none in the non-SBI group, with a significant difference (*P* = 0.018) (Table 1).

**Table 1:** Clinical characteristics, vital signs, and blood laboratory findings among febrile infants in SBI and non-SBI groups aged 91 to 120 days

	Number (%) or Median (Q1, Q3)						
Variables	SBI (*n* = 119)		Non-SBI (*n* = 145)			*P*-value	
Age(d)	106.0 (99.0, 113.0)	105.0 (100.0, 114.0)					0.829
Male sex, %	92 (77.3%)	72 (49.7%)					< 0.001
GA (wk)	38.5 (37.5, 39.5)	38.0 (37.0, 39.0)					0.831
Birth weight (g)	3035.0 (2870.0, 3290.0)	3000.0 (2700.0, 3250.0)					0.141
Admission weight (g)	6600.0 (6000.0, 7000.0)	6000.0 (5600.0, 6850.0)					0.001
BT at triage (°C)	38.7 (38.0, 39.5)	38.5 (38.0, 38.7)					0.002
Pulse rates at triage (/min)	169.0 (154.0, 188.0)	168.0 (156.0, 183.0)					0.958
Highest BT (°C)	38.9 (38.2, 39.6)	38.5 (38.1, 38.9)					< 0.001
Highest BT of hospital stay (°C)	38.7 (38.2, 39.2) (*n* = 63)^*^	38.6 (38.4, 39.1) (*n* = 57)^*^					0.575
Fever subsidence time (d)†	2.0 (1.0, 2.0)	2.0 (1.0, 3.0)					0.353
Duration of hospitalization (days)	3.0 (3.0, 4.0)	3.0 (2.0, 4.0)					0.006
Laboratory test (blood)							
WBC (/uL)/1000	15.5 (10.9, 19.7)	11.2 (7.5, 15.3)					< 0.001
Neutrophils (%)	51.5 (40.9, 60.2)	43.3 (34.6, 56.2)					< 0.001
Bands (%)	0.4 (0.0, 0.0)	0.0 (0.0, 0.0)					0.900
CRP (mg/dl)	2.6 (1.0, 4.7)	0.6 (0.2, 1.4)					< 0.001
Blood culture (+)(%)	5 (4.2)	0 (0)					0.018

SBI, severe bacterial infection; BT, body temperature; CRP, C-reactive protein; GA, gestational age; WBC, white blood cells

∗Data collected in patients who still had a body temperature of > 38 °C after admission

† Defined as the days during which no fever episode was detected for a consecutive 48-h period

Logistic regression analysis revealed that male sex, higher admission weight, elevated temperature at triage, higher peak temperature, increased WBC count, higher neutrophil count, and elevated CRP levels were all associated with a greater likelihood of SBI. After adjusting for other variables, only higher peak temperature, elevated WBC count, and increased CRP levels remained significantly associated with SBI (Table 2).

**Table 2:** Revealing factors linked to SBI through logistic regression analysis

		Univariate analysis				Multivariable analysis (adjusted)		
Variables	*N* (%) or Median(Q1, Q3)	OR	95% C.I.	*P*-value		OR	95% C.I.	*P*-value
Gender								
Female	27 (27.0%)	1.000				1.000		
male	92 (56.1%)	3.455	2.016–5.920	< 0.001		3.675	2.001–6.749	< 0.001
Admission weight (kg)	6.6 (6.0, 7.0)	1.300	1.016–1.665	0.037				
BT at triage (°C)	38.7 (38.0, 39.5)	1.670	1.213–2.299	0.002				
Highest BT (°C)	38.9 (38.2, 39.6)	1.809	1.313–2.492	< 0.001		1.864	1.286–2.702	0.001
Laboratory test (blood)								
WBC (/uL)/1000	15.5 (10.9, 19.7)	1.116	1.066–1.169	< 0.001		1.099	1.043–1.159	< 0.001
Neutrophils (%)	51.5 (40.9, 60.2)	1.028	1.011–1.045	0.001				
CRP (mg/dL)	2.6 (1.0, 4.7)	1.235	1.116–1.367	< 0.001		1.135	1.021–1.261	0.019

BT, body temperature; CRP, C-reactive protein; WBC, white blood cell; OR, odds ratio

The risk of SBI in male infants was 3.675 times greater than in female infants (95% CI: 2.001–6.749, *P* < 0.001). Furthermore, higher maximum body temperature was linked to an increased risk of SBI (Odds Ratio = 1.864, 95% CI = 1.286–2.702, *P* = 0.001). For every 1 °C rise in body temperature at triage, the risk of SBI in infants increased by 86.4%. Elevated WBC counts and CRP levels were also associated with a higher risk of SBI in young infants. For every 1000/µL increase in WBC count, the risk of SBI increased by 9.9%, while each 1 mg/L increase in CRP levels was associated with a 13.5% higher risk of SBI.

Infants with SBI displayed distinct clinical traits based on their fever status during triage. Regardless of their fever status at triage, infants with SBI were more likely to be male, have higher admission body weight, and exhibit elevated CRP levels (all *P* < 0.05). Febrile infants with SBI at triage showed further differences, including higher BT, extended hospital stays, elevated WBC counts, increased neutrophil percentages, and a higher incidence of positive blood culture growth (Table 3).

**Table 3:** Clinical findings and laboratory results in young infants aged 91–120 days within the SBI and Non-SBI groups: a distinction between fever and non-fever at triage

	*N* (%) or Median (Q1, Q3)								
	**Body temperature < 38**°C					**Body temperature ≥ 38**°C			
**Variables**	SBI (*N* = 24)	Non-SBI (*N* = 29)	***P*** **-value**				SBI (*N* = 95)	Non- SBI (*N* = 116)	***P*** **-value**
Male sex, %	20 (83.3%)	16 (55.2%)	< 0.001				72 (75.8%)	56 (48.3%)	< 0.001
Fever time									
No fever	16 (66.7)	25 (86.2)	0.091				0 (0)	0 (0)	
Before PER	0 (0)	0 (0)					95 (100)	116 (100)	
After triage	8 (33.3)	4 (13.8)					0 (0)	0 (0)	
GA (wk)	38.0 (37.0, 40.0)	38.5 (37.0, 40.0)	0.776				38.0 (37.0, 39.0)	38.0 (37.0, 39.0)	0.706
Birth weight (g)	3065.0 (2880.0, 3400.0)	3000.0 (2740.0, 3345.0)	0.344				3015.0 (2870.0, 3260.0)	3000.0 (2680.0, 3250.0)	0.238
Admission weight (g)	6850.0 (6215.0, 7300.0)	6200.0 (5900.0, 6800.0)	0.024				6400.0 (6000.0, 7000.0)	6000.0 (5505.0, 6900.0)	0.005
BT at triage (°C)	37.70 (37.20, 37.70)	37.6 (37.20, 37.70)	0.869				39.10(38.60, 39.60)	38.60(38.20, 38.90)	< 0.001
Pulse rates at triage (/min)	157.0 (141.5, 169.0)	153.0 (145.0, 170.0)	0.879				171.5 (159.0, 189.0)	170.0 (160.0, 186.5)	0.868
Highest BT (°C)	37.70 (37.60, 38.10)	37.60 (37.20, 37.70)	0.206				39.30 (38.60, 39.70)	38.60 (38.30, 39.10)	< 0.001
Highest BT of hospital stay (°C)	38.20 (38.10, 38.60)	38.50 (38.30, 39.40)	0.305				38.90 (38.50, 39.40)	38.60 (38.40, 39.10)	0.261
Fever subsidence time (d)^∗^	2.0 (2.0, 2.0)	3.0 (2.5, 4.0)	0.032				2.0 (1.0, 2.0)	2.0 (1.0, 3.0)	0.392
Duration of hospitalization(days)	3.0 (2.0, 3.5)	3.0 (2.0, 4.0)	0.591				3.0 (3.0, 4.0)	3.0 (2.0, 3.0)	0.001
Laboratory test (blood)									
WBC (/uL)	16400.0 (11400.0, 19400.0)	11400.0 (9000.0, 15400.0)	0.080				15500.0 (10800.0, 19700.0)	11100.0 (7300.0, 15300.0)	< 0.001
Neutrophils (%)	43.40 (31.70, 59.30)	39.90 (22.60, 51.70)	0.226				52.00 (42.10, 60.90)	43.60 (35.20, 56.90)	0.001
Bands (%)	0.0 (0.0, 0.0)	0.0 (0.0, 0.0)	0.365				0.0 (0.0, 0.0)	0.0 (0.0, 0.0)	0.273
CRP (mg/dL)	2.2 (0.8, 5.1)	0.9 (0.3, 1.4)	0.012				2.7 (1.2, 4.6)	0.6 (0.2, 1.4)	< 0.001
Blood culture (+)(%)	1 (4.2)	0 (0)	0.453				4 (4.2)	0 (0)	0.040

BT, body temperature; CRP, C-reactive protein; GA, gestational age; WBC, white blood cells

^*^Defined as the date during which no fever episode was detected for a consecutive 48-h period

**Fig. 1:**
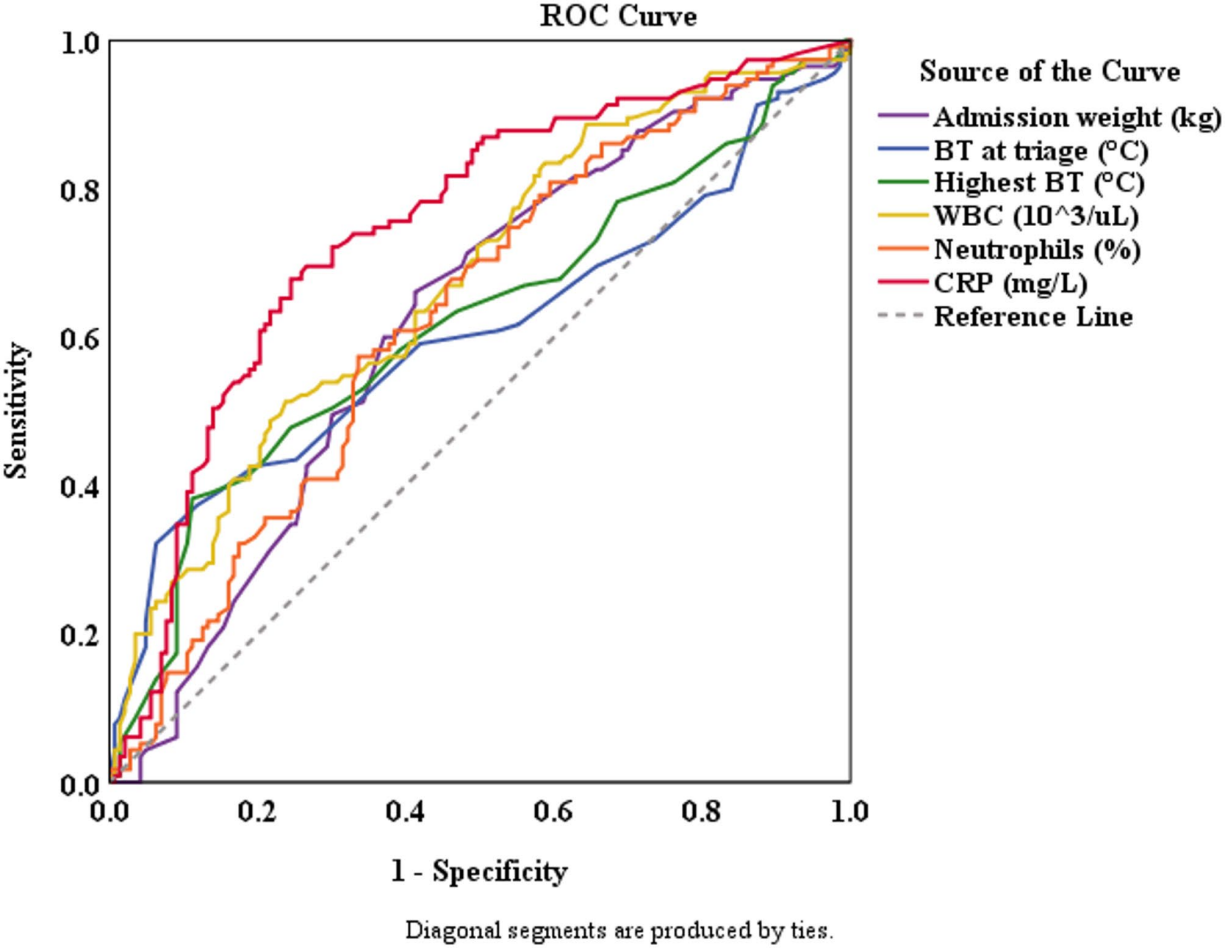
ROC for variables associated with SBI. Area under the curve for admission body weight is 0.623 (95% CI: 0.554, 0.691); for BT at triage is 0.603 (95% CI: 0.531, 0.675); for highest BT is 0.619 (95% CI: 0.549, 0.689); for WBC is 0.674 (95% CI: 0.609, 0.740); for ANC is 0.628 (95% CI: 0.560, 0.696); and for CRP is 0.747 (95% CI: 0.5686, 0.808)

The diagnostic accuracies for predicting SBI using the various cutoff values are shown in Table 4; Fig. 1. An admission body weight ≥ 6.2 kg exhibited a sensitivity of 66.1% and a specificity of 58.7%, with an AUC of 0.623. A triage BT ≥ 39.3 °C demonstrated a sensitivity of 32.2% and a specificity of 93.7%, with an AUC of 0.603. Peak BT (≥ 39.4 °C) showed a sensitivity of 38.3% and a specificity of 88.8%, with an AUC of 0.619. WBC counts ≥ 15,500/µL indicated a sensitivity of 51.3% and a specificity of 76.2%, with an AUC of 0.674. Neutrophil percentages ≥ 49.4% exhibited a sensitivity of 57.4% and a specificity of 66.4%, with an AUC of 0.628. Finally, CRP levels ≥ 1.44 mg/dL demonstrated a sensitivity of 67.8% and a specificity of 75.5%, with an AUC of 0.747.

**Table 4:** Receiver operating characteristic curve analysis of factors for the prediction of SBI in infant aged 91 ∼ 120 days

	Criterion values and coordinates of ROC curve								Area under the ROC curve					
Variables	Value	Sensitivity	Specificity	PPV	NPV	+LR	-LR		Area	SE	95% C.I.			*P*-value
Admission weight (kg)	≥ 6.2	0.661	0.587	0.568	0.679	1.602	0.567		0.623	0.035	0.554	-	0.691	**0.001**
BT at triage (°C)	≥ 39.3	0.322	0.937	0.808	0.627	5.112	0.724		0.603	0.037	0.531	-	0.675	**0.004**
Highest BT (°C)	≥ 39.4	0.383	0.888	0.737	0.637	3.420	0.695		0.619	0.036	0.549	-	0.689	**0.001**
WBC (ul)/1000	≥ 15.5	0.513	0.762	0.639	0.656	2.158	0.639		0.674	0.033	0.609	-	0.740	**< 0.001**
Neutrophil (%)	≥ 49.4	0.574	0.664	0.584	0.655	1.710	0.641		0.628	0.035	0.560	-	0.696	**< 0.001**
CRP (mg/dL)	≥ 1.44	0.678	0.755	0.695	0.741	2.771	0.426		0.747	0.031	0.686	-	0.808	**< 0.001**

BT, body temperature; CRP, C-reactive protein; LR, likelihood ratio

The results of the receiver operating characteristic (ROC) analysis for predicting SBI in young febrile infants are summarized in Table 5. The following criteria demonstrated 100% sensitivity for excluding pediatric SBI: admission body weight ≤ 2.5 kg, BT ≤ 36.8 °C, WBC count ≤ 140/µL, neutrophil percentage ≤ 5.1%, and CRP levels ≤ 0.1 mg/dL. In contrast, the criteria showing 100% specificity for detecting the condition included: admission body weight ≥ 11.4 kg, BT ≥ 40.5 °C, WBC count ≥ 36,300/µL, neutrophil percentage ≥ 81.9%, and CRP levels ≥ 28.2 mg/dL. The pathogens associated with SBI include *Escherichia coli (E. coli)*, *Klebsiella pneumoniae (KP)*, *Enterococcus faecalis*, *Proteus mirabilis*, *Citrobacter freundii*,* Citrobacter koseri*,* Pseudomonas aeruginosa*, Gram-negative bacilli (GNB), and *Klebsiella oxytoca* in urinary tract infections. Additionally, *E. coli* and *Group B Streptococcus (GBS)* were identified in blood culture infections. *E. coli* was the most prevalent potential pathogen in infants with SBI, accounting for 104 UTI cases and 2 cases of bacteremia. No cases of bacterial meningitis were reported. Viral infections were identified in 26 cases, with influenza in 10 cases, RSV in 9 cases, enterovirus in 4 cases, and parainfluenza in 3 cases. Notably, no viral co-infections were observed in conjunction with SBI in this study.

**Table 5:** Cutoff value in sensitivity = 1.0 or specificity = 1.0 for the prediction of SBI

Variables	Cutoff	Sensitivity	Specificity	PPV	NPV	+LR	-LR
Admission weight (kg)	2.50	1.000	0.000	0.451	-	1.000	-
	11.40	0.000	1.000	-	0.549	-	1.000
Body temperature (°C)	36.8	1.000	0.007	0.452	1.000	1.007	0.000
	40.5	0.009	1.000	1.000	0.551	-	0.991
WBC (uL)	140	1.000	0.000	0.451	-	1.000	-
	36,300	0.009	1.000	1.000	0.551	-	0.991
Neutrophil (%)	5.1	1.000	0.000	0.451	-	1.000	-
	81.9	0.017	1.000	0.554	0.936	-	0.983
CRP (mg/dl)	< 0.1	1.000	0.000	0.451	-	1.000	-
	28.2	0.000	1.000	-	0.549	-	1.000

## Discussion

Fever in young infants often raises significant concerns among families. This worry, often fueled by fear, typically leads to visits to the emergency department (ED) [17]. In the PED, vital signs and blood tests are rapid parameters that help doctors determine whether to rule in or rule out SBI. In our study conducted in the PED, we found that clinical and laboratory factors, such as male sex, and elevated body temperature(BT), increased white blood cell (WBC) counts, higher neutrophil percentages, and elevated C-reactive protein (CRP) levels, may serve as significant predictors of SBI in febrile infants aged 91–120 days. These findings provide valuable insights for clinicians, enabling them to identify high-risk infants more effectively and ensure that appropriate treatment is initiated without delay, thereby minimizing the risk of complications.

Although no studies have specifically focused on infants aged 91–120 days, research on other age groups of young infants with SBI can provide a basis for comparison. Our study demonstrated that male infants aged 91–120 days had a higher incidence of SBI. Similarly, previous studies have shown that among infants younger than 60 days, the prevalence of SBI is higher in boys than in girls [18, 19]. The differences may be attributed to anatomical factors, such as higher UTI rates in male infants due to lack of circumcision, and genetic factors, as females have two X chromosomes, providing greater genetic diversity and a lower likelihood of X-linked primary immunodeficiencies, which can cause recurrent bacterial, fungal, and viral infections [20]. The influence of sex steroids on autoimmune diseases and susceptibility to infections typically becomes evident after puberty [21]. Previous studies have indicated that when considering vital signs, BT in febrile infants younger than 60 days is closely linked to the risk of SBI [19]. Establishing a BT threshold to predict SBI would be valuable for clinicians. For example, one study in infants under 3 months found that a BT ≥ 38.5 °C was a significant predictor of SBI [22]. Another cohort study focusing on infants under 6 months showed that fever > 39 °C was associated with an increased risk of SBI [23]. Additionally, a systematic review reported a higher risk of SBI with fever > 40 °C [24]. Furthermore, in a prospective series, temperatures > 41 °C were associated with almost 50% of children being found to have an SBI [25]. Our study found that febrile infants with bacteremia had elevated BT at triage and peak values, with BT ≥ 39.3 °C at triage and ≥ 39.4 °C at peak, showing an AUC > 0.6 for SBI risk. A higher BT in the PED strongly predicted SBI.

Routine blood tests revealed a significantly higher WBC count, neutrophil percentage, and CRP protein level in the SBI group. Previous studies in various age groups have yielded similar findings, with elevated WBC counts and higher neutrophil levels frequently observed in SBI cases [26, 27]. Similarly, numerous studies involving infants aged < 6 months demonstrated higher CRP levels in patients with SBI [16, 27]. The AUC represents the ability of a specific test or biomarker to distinguish between infants with and without SBI. In our study, WBC count ≥ 15,500/µL corresponded to an AUC of 0.674, neutrophil percentages > 49.4% yielded an AUC of 0.628, and CRP levels > 1.44 mg/dL resulted in an AUC of 0.747. In a study of infants aged 1–36 months, AUCs of 0.761 for WBC > 15,000/µL, 0.805 for absolute neutrophil count(ANC) > 10,200/µL, and 0.905 for CRP > 7 mg/dL were reported [26] Another study involving infants under 90 days showed AUCs of 0.632 for WBC > 13,830/µL, 0.681 for ANC > 6,370/µL, and 0.815 for CRP > 7.2 mg/dL [27]. Among the predictive indicators for SBI, CRP demonstrated the best discriminatory value, consistently yielding higher AUCs than WBC and ANC, and outperforming WBC and neutrophil percentages as predictive markers.

In clinical practice, diagnostic tests for SBI are often categorized into three zones: high sensitivity, high specificity, and indeterminate. Our study identified specific cut-off points for various biomarkers to aid in the diagnosis of SBI in febrile infants admitted to the PED. We found that serum parameters were highly valid for diagnosing SBI when WBC counts were ≥ 36,300/µL, neutrophil counts were ≥ 81.9%, or CRP levels were ≥ 28.2 mg/dL in febrile infants. Conversely, for ruling out SBI, WBC counts of < 14,000/µL, neutrophil counts of < 5.1%, and CRP levels of < 0.1 mg/dL were effective. Additionally, infants were more likely to have SBI when their BT exceeded 40.5 °C, while SBI could be ruled out when their BT was below 36.8 °C. These findings suggest that specific serum parameters and BT measurements can serve as valuable indicators for diagnosing or ruling out bacteremia in febrile infants, thereby assisting clinicians in making informed decisions.

Identifying the etiology of SBI is crucial for clinicians to determine appropriate treatment. Among the 119 cases of SBI in our study, urinary tract infection (UTI) was the most common cause, accounting for 95.8% (114/119), followed by bacteremia, which accounted for 4.2% (5/119). Similarly, another review and cohort study identified UTI as the leading cause of SBI, followed by sepsis, pneumonia, cellulitis, abscesses, and meningitis [28, 29]. Although Watson et al. found a higher incidence of bacteremia in infants under one year of age [30], the widespread use of vaccines has contributed to a greater than 50% reduction in bacteremia caused by *Streptococcus pneumoniae* and *Haemophilus influenzae type B* [28, 31]. Another study in Taiwan also showed that the incidence of bacteremia and SBI in young infants declined notably following the introduction of the *Haemophilus influenzae type b*(Hib) and PCV vaccines [29].

Bacterial meningitis affects the membranes surrounding the brain and causes severe morbidity and mortality in children. No cases of bacterial meningitis were observed in our study. A recent study on the evolving trends in bacterial meningitis among children revealed a decline in prevalence across age groups and a reduction in the total number of cases in recent years [32]. After the introduction of conjugated pneumococcal vaccines, the incidence of *E. coli*,* S. aureus* and Salmonella spp. increased, whereas that of *S. pneumoniae* decreased in children aged 3 months to 3 years [33]. These shifts are attributed to factors including widespread vaccination, prenatal maternal vaginal bacterial screening, the establishment of national health insurance, improvements in antibiotic treatments, and new approaches to patient care.

This study has several limitations. Firstly, its retrospective design not only made it difficult to establish causal relationships between risk factors and SBI but also limited the availability of procalcitonin levels for SBI prediction. Secondly, as the study was conducted at a single medical center in Taiwan, the findings may not be widely applicable. Lastly, some potential risk factors for SBI, such as the duration of fever before presenting to the PED, were not considered in the analysis.

## Conclusion

Clinical signs, such as BT at triage, peak BT, and blood laboratory results, can provide important clues for diagnosing SBI in infants aged 90–120 days. Higher BT at triage, elevated WBC counts, increased neutrophil percentages, and raised CRP levels may help predict the likelihood of SBI in febrile infants aged 91–120 days admitted to the PED.

## Data Availability

The datasets used and analyzed during the current study are available from the corresponding author on reasonable request.
